# Tamoxifen use reduces the risk of osteoporotic fractures in women with breast cancer in Asia: a nationwide population-based cohort study

**DOI:** 10.1186/s12891-015-0580-8

**Published:** 2015-05-20

**Authors:** Huey-En Tzeng, Chih-Hsin Muo, Hsien-Te Chen, Wen-Li Hwang, Horng-Chang Hsu, Chun-Hao Tsai

**Affiliations:** Graduate Institute of Clinical Medicine, China Medical University, #91 Hsueh-Shih Road, Taichung, 404 Taiwan; Division of Hematology/Oncology, Taichung Veterans General Hospital, Taichung, Taiwan; School of Medicine, China Medical University, Taichung, Taiwan; Management Office for Health Data, China Medical University Hospital, Taichung, Taiwan; Department of Orthopedics, China Medical University Hospital, Taichung, Taiwan; School of Chinese Medicine, China Medical University, Taichung, Taiwan

**Keywords:** Breast cancer, Fracture, Risk, Tamoxifen, Population-based cohort study

## Abstract

**Purpose:**

Bone mineral density changes with tamoxifen treatment have been reported in pre- and post-menopausal women with breast cancer. However, there remains controversy as to whether tamoxifen significantly reduces fracture rates in different age groups. Breast cancer occurs at 10-20 years younger in Asian women compared with Western women. Therefore we conducted this population-based case-control study to determine whether or not tamoxifen use is associated with osteoporotic fractures.

**Patients and methods:**

We selected 75488 women with breast cancer with no prior history of fractures from the Longitudinal Health Insurance Database for Catastrophic Illness Patients in 2000-2011. They were followed from the date of the diagnosis of breast cancer to the date a hip, vertebral or wrist fracture occurred. Because the use of tamoxifen was a time-dependent variable, we used a Cox proportional hazard model with time-dependent exposure covariates to estimate the risk of a fracture.

**Results:**

There were 50257 and 25231 women with breast cancer who did and did not receive tamoxifen treatment, respectively. The tamoxifen users had lower risks for overall fractures with hazard ratios (HRs) of 0.52 and 0.59 in the crude and adjusted models (95 % CI = 0.45-0.61 and 0.51-0.69), respectively. They also had lower risks for hip (HR = 0.55, 95 % CI = 0.45-0.67) and vertebral (HR = 0.64, 95 % CI = 0.50-0.82) fractures in the adjusted model. The risk of fractures decreased with an increasing dosage of tamoxifen. Regardless of the age group, the tamoxifen users had a lower risk of fractures than the non-users.

**Conclusion:**

In this Asian population-based case-control study, tamoxifen use was associated with a reduction in osteoporotic fractures, especially in hip fractures.

## Background

Breast cancer survivors have an increased risk of osteoporosis and fractures [1, 2]. This elevated risk of fracture could be due to the effects of chemotherapy, ovarian failure, early menopause, and the use of aromatize inhibitors (AI) [3–5]. Although the peak in the age of breast cancer among Asian women occurs much earlier than in Western women (40-50 years vs. 60-70 years) [6], we previously showed that Asian breast cancer survivors have a higher risk of fractures, particularly those below the age of 50 [7].

Tamoxifen has been used since the 1990s as an adjuvant treatment for breast cancer patients who are estrogen receptor (ER)-positive. Tamoxifen has estrogen-like effects on bone metabolism that may result in an increase and stabilization of BMD. However, the effectiveness of this bone protection between pre- and post-menopausal women remains debatable. A significant loss of BMD in pre-menopausal women receiving Tamoxifen treatment has been reported, whereas it has been shown to prevent bone loss in post-menopausal women [8]. In addition, the proactive effect in terms of fractures related to tamoxifen is also controversial in clinical practice. Kristensen et al. showed that tamoxifen offered no further protection against fractures in old age and may even increase the risk of fractures at particular sites [9]. On the other hand, a large population-based study in Canada revealed a protective effect of concurrent tamoxifen treatment against overall osteoporotic fractures, especially hip fractures [10].

Whether or not tamoxifen offers a protective effect with regards to the risk of fractures in Asian women with breast cancer should ideally be investigated among those whose major peak age is 10 years younger than in Western women with breast cancer. Therefore, we used National Health Insurance (NHI) claims data in Taiwan to assess this relationship.

## Methods

We used the Longitudinal Health Insurance Database for Catastrophic Illness Patients (LHID-CIP), a part of the National Health Insurance Research Database (NHIRD), from the National Health Research Institutes (NHRI) for this retrospective cohort study. The Taiwan Bureau of National Health Insurance (TBNHI) established a universal NHI Program in 1995, which currently covers over 99 % of the population in Taiwan. The TBNHI entrusted the NHRI to construct and maintain the NHIRD for research purposes. The NHIRD includes all inpatient and outpatient claims for each beneficiary from 1997 to 2011. The LHID-CIP includes all patients conforming to the guidelines of the TBNHI for a catastrophic illness certificate. Patients with a catastrophic illness certificate are exempt from copayment for medical care, and this certificate is formally reviewed by a physician upon application. Diseases are, identified in the NHIRD based on International Classification of Diseases, Ninth Revision, Clinical Modification (ICD-9-CM) codes. Based on the Personal Information Protection Act, the identification of beneficiary is scrambled by the TBNHI, and all researchers are required to sign a written agreement regarding their intentions when attempting to obtain the private data of patients when the NHIRD is released. This study was also approved by the Institutional Review Board of China Medical University Hospital.

We collected data on women with a diagnosis of breast cancer (ICD-9-CM 174) from 2000 to 2011 from the LHID-CIP as the study population, and the date of diagnosis was used as the index date. However ,the data dose not record the stage of breast cancer. Those that received tamoxifen [Anatomical Therapeutic Chemical code (ATC code) L02BA01] before the index date or those with a prior history of fractures (ICD-9-CM 800-829) within 1 year before the index date were excluded. All of the included breast cancer patients were followed from the index date until the date a hip (ICD-9-CM 820), vertebral (ICD-9-CM 805) or wrist (ICD-9-CM 815) fracture occurred. Those without a fracture were followed to the date of withdrawal from the NHI program or to the end of 2011.

The exposure was defined the breast cancer women received Tamoxifen during the study period. According to the World Health Organization defined the assumed average maintenance dose per day for a drug used for its main indication in adults, defined daily doses (DDD) is a statistical measure of drug consumption. (WHO Collaborating Centre for Drug Statics Methodology. Available from: http://www.whocc.no/use_of_atc_ddd/#Pharmaceutical). The accumulated Tamoxifen DDDs during the study period was calculated for each user.

The comorbidities considered were hypertension (ICD-9-CM 401-405), hyperlipidemia (ICD-9-CM 272), diabetes (ICD-9-CM 250), peripheral artery disease (PAD, ICD-9-CM 440.0, 440.2, 440.3, 440.8, 440.9, 443, 444.0, 444.22, 444.8, 447.8 and 447.9), stroke (ICD-9-CM 430-438) and osteoporosis (ICD-9-CM 733.0 and 733.1). All comorbidities were defined before the index date. The medical treatments considered were hormone replacement therapy (HRT, ATC codes G03AA08, G03AC06, G03CA57, G03DA02, G03DA03, G03DA04, G03FA01, G03FA02, G03FA12, G03FB01, G03FB06 and L02AB02) and aromatase inhibitors (AIs, ATC code L02BG). HRT users were defined as those who received treatment before the index date. Because AIs are used to treat women with breast cancer, AI users were defined as those who received treatment before the fracture occurred.

The chi-square test was used to test differences between age groups (<50, 50-59, 60-69 and ≥70 years), and comorbidities between the women with breast cancer who did and did not receive tamoxifen treatment. Because the breast cancer patients may not always have received tamoxifen and AIs during the study period and this may have overestimated the effect of the drugs, we used a Cox proportional hazard model with time-dependent exposure covariates to estimate the risk of fractures in order to reduce this bias. The multivariate Cox proportional hazard model with time-dependent covariates was adjusted for age, monthly income, comorbidities and medicine used, and significant differences were found (Table 1). The effects of dose on fractures were estimated according to the dosage or duration of tamoxifen treatment. The association between different locations of fractures and tamoxifen use was then assessed. The age-specific risk and each comorbidity-specific risk for different locations of fractures among those who did and did not receive tamoxifen were also estimated. All statistical analyses were performed using SAS software version 9.3 (SAS Institute Inc., Carey, NC), and a two-tailed p value of less than 0.05 was considered to indicate statistical significance.

**Table 1:** Demographic characteristics of the study subjects

	All N = 75488		With tamoxifen N = 50257		Without tamoxifen N = 25231		
Variable	n	%	n	%	n	%	p-value
Age, years							<0.0001
<50	35715	47.3	25778	51.3	9937	39.4	
50-59	21676	28.7	13124	26.1	8552	33.9	
60-69	11353	15.0	7001	13.9	4352	17.6	
70+	6744	8.93	4354	8.66	2390	9.47	
Urbanization							0.34
Urban	50793	67.3	33874	67.4	16919	67.1	
Rural	24695	32.7	16383	32.6	8312	32.9	
Month income, NTD							<0.0001
<= 15000	12795	17.0	8231	16.4	4564	18.1	
15001-20000	39463	52.3	26326	52.4	13137	52.1	
>20000	33230	30.8	15700	31.2	7530	29.8	
Comorbidity							
Hypertension	21882	29.0	14142	28.1	7740	30.7	<0.0001
Hyperlipidemia	15458	20.5	9952	19.8	5506	21.8	<0.0001
Diabetes	8948	11.9	5698	11.3	3250	12.9	<0.0001
PAD	1477	1.96	905	1.80	572	2.27	<0.0001
Stroke	1715	2.27	1012	2.01	703	2.79	<0.0001
Osteoporosis	8024	10.6	5114	10.2	2910	11.5	<0.0001
Medicine, n (%)							
HRT used before the index date	36690	48.6	24487	48.7	12203	48.4	0.35
Aromatase inhibitors used before the end point	20507	27.2	16273	32.4	4234	16.8	<0.0001

Chi-square test

PAD, peripheral artery disease

## Results

We collected data on 75,488 women with breast cancer, of whom 50,257 and 25,231 did and did not receive tamoxifen treatment, respectively. Compared with the patients who did not receive tamoxifen treatment, the tamoxifen users were younger (mean age 51.7 vs. 53.5 years, data not shown) and had fewer comorbidities (Table 1). There was no significant difference in the urbanization of residential area between the tamoxifen users and non-users, however the tamoxifen users had a higher income than the non-users.

According to the Cox proportional hazard model with time-dependent covariates, the tamoxifen users had lower risks of fractures with hazard ratios (HRs) of 0.52 and 0.59 in the crude and adjusted models (95 % CI = 0.45-0.61 and 0.51-0.69), respectively (Table 2). The same trends for hip, vertebral and wrist fractures were also noted, however only wrist fractures were found to not be significantly different. The risk of fractures significantly decreased with the dosage and duration of tamoxifen use for both overall and hip fractures.

**Table 2:** Hazard ratios (HRs) and 95 % confidence intervals (CIs) for different locations of fractures in the time-dependent model

Location	Crude HR (95 % CI)		p-value	Adjusted HR (95 % CI)		p-value
Overall						
Tamoxifen use (yes vs. no)	0.52	(0.45-0.61)	<0.0001	0.59	(0.51-0.69)	<0.00001
Increased dose of tamoxifen, per 100 DDD	0.98	(0.97-0.99)	0.0003	0.98	(0.97-0.99)	0.0002
Increased duration of tamoxifen use, per 100 days	0.98	(0.97-0.99)	0.0004	0.98	(0.97-0.99)	0.0002
Hip fracture						
Tamoxifen use (yes vs. no)	0.48	(0.39-0.57)	<0.0001	0.55	(0.45-0.67)	<0.0001
Increased dose of tamoxifen, per 100 DDD	0.97	(0.96-0.99)	0.0003	0.97	(0.96-0.99)	0.0005
Increased duration of tamoxifen use, per 100 days	0.98	(0.96-0.99)	0.001	0.97	(0.96-0.99)	0.0004
Vertebral fracture						
Tamoxifen use (yes vs. no)	0.59	(0.46-0.75)	<0.0001	0.64	(0.50-0.82)	0.0003
Increased dose of tamoxifen, per 100 DDD	0.98	(0.97-1.00)	0.15	0.98	(0.97-1.00)	0.06
Increased duration of tamoxifen use, per 100 days	0.98	(0.97-1.00)	0.09	0.98	(0.97-1.00)	0.06
Wrist fracture						
Tamoxifen use (yes vs. no)	0.86	(0.43-1.71)	0.66	0.84	(0.42-1.68)	0.62
Increases dose of tamoxifen, per 100 DDD	1.01	(0.96-1.06)	0.83	1.01	(0.96-1.06)	0.76
Increased duration of tamoxifen use, per 100 days	1.01	(0.96-1.05)	0.83	1.01	(0.96-1.05)	0.86

Manually adjusted for tamoxifen use, age, monthly income, comorbidity and aromatase inhibitor use before the end point

DDD, defined daily dose

The associations between fractures and potential risk factors are presented in Table 3. In the multivariate Cox proportional hazard model with time-dependent covariates, the risk of fractures increased with age. Compared with the patients who had a higher monthly income (>20000 NTD), those with a lower monthly income had a higher risk of fractures. The patients with diabetes had the highest risk (HR = 1.59, 95 % CI = 1.37-1.85), followed by stroke, osteoporosis and hypertension. However, the patients with hyperlipidemia and AI treatment had a significantly lower risk of fractures (HR = 0.83 and 0.80, 95 % CI = 0.72-0.96 and 0.68-0.95, respectively).

**Table 3:** Hazard ratios (HRs) and 95 % confidence intervals (CIs) for fractures and potential risk factors in the time-dependent model

Variable	Crude HR (95 % CI)		p-value	Adjusted HR (95 % CI)		p-value
Age, years	1.10	(1.10-1.11)	<0.0001	1.09	(1.08-1.10)	<0.0001
Monthly income, NTD						
<= 15000	4.85	(3.98-5.91)	<0.0001	1.34	(1.08-1.66)	0.009
15001-20000	2.25	(1.86-2.71)	<0.0001	1.51	(1.25-1.82)	<0.0001
>20000	1.00	Ref.		1.00	Ref.	
Comorbidity (yes vs. no)						
Hypertension	4.48	(3.94-5.09)	<0.0001	1.27	(1.09-1.48)	0.002
Hyperlipidemia	2.19	(1.92-2.50)	<0.0001	0.83	(0.72-0.96)	0.01
Diabetes	3.65	(3.18-4.19)	<0.0001	1.59	(1.37-1.85)	<0.0001
PAD	2.74	(2.00-3.74)	<0.0001	0.99	(0.72-1.36)	0.96
Stroke	5.40	(4.27-6.83)	<0.0001	1.38	(1.08-1.76)	0.01
Osteoporosis	3.11	(2.70-3.58)	<0.0001	1.38	(1.19-1.59)	<0.0001
Aromatase inhibitor use before the end point	1.18	(1.00-1.40)	0.049	0.80	(0.68-0.95)	0.01

Manually adjusted for tamoxifen use, age, monthly income, comorbidity and aromatase inhibitor use before the end point

PAD, peripheral artery disease

Table 4 shows the age-specific risk of fractures among those who did and did not receive tamoxifen treatment. In each age group, the risks of overall and hip fractures were lower in the tamoxifen users than in the non-users. For vertebral fractures, only the tamoxifen users who were 70 years and older had a significantly lower risk of fractures. Comorbidity-stratified analysis is shown in Table 5. Regardless of the comorbidities, the tamoxifen users tended to have a lower risk of fractures than the non-users.

**Table 4:** Adjusted hazard ratios (HRs) and 95 % confidence intervals (CIs) for different fracture locations in the time-dependent model among age group

Age group	Tamoxifen user vs. non-user HR (95 % CI)		p-value	Interaction p
Overall				0.29
<50	0.54	(0.36-0.82)	0.004	
50-59	0.58	(0.40-0.83)	0.003	
60-69	0.66	(0.50-0.88)	0.005	
70+	0.59	(0.48-0.74)	<0.0001	
Hip fracture				0.046
<50	0.39	(0.19-0.81)	0.01	
50-59	0.38	(0.20-0.72)	0.003	
60-69	0.56	(0.38-0.81)	0.002	
70+	0.61	(0.47-0.78)	0.0001	
Vertebral fracture				0.86
<50	0.59	(0.34-1.03)	0.06	
50-59	0.74	(0.46-1.19)	0.21	
60-69	0.84	(0.54-1.32)	0.46	
70+	0.54	(0.34-0.85)	0.008	
Wrist fracture				0.26
<50	0.90	(0.31-2.57)	0.84	
50-59	0.76	(0.25-2.34)	0.63	
60-69	1.44	(0.20-10.6)	0.72	
70+	1.10	(0.07-17.9)	0.94	

Manually adjusted for monthly income, comorbidity and aromatase inhibitor use before the end point

**Table 5:** Hazard ratio (HRs) and 95 % confidence intervals (CIs) for fractures in the tamoxifen users compared with non-users using the time-dependent model stratified by comorbidity

Comorbidity	Tamoxifen user vs. non-user HR (95 % CI)	p-value	Interaction p
Hypertension			0.18
No	0.67 (0.53-0.85)	0.001	
Yes	0.55 (0.46-0.66)	<0.0001	
Hyperlipidemia			0.97
No	0.60 (0.50-0.72)	<0.0001	
Yes	0.58 (0.45-0.74)	<0.0001	
Diabetes			0.56
No	0.62 (0.52-0.74)	<0.0001	
Yes	0.54 (0.41-0.71)	<0.0001	
PAD			0.58
No	0.59 (0.50-0.68)	<0.0001	
Yes	0.73 (0.37-1.46)	0.38	
Stroke			0.32
No	0.59 (0.50-0.69)	<0.0001	
Yes	0.68 (0.41-1.13)	0.14	
Osteoporosis			0.33
No	0.61 (0.51-0.73)	<0.0001	
Yes	0.54 (0.40-0.72)	<0.0001	

PAD, peripheral artery disease

Manually adjusted for monthly income, comorbidity and aromatase inhibitor use before the end point

## Discussion

Tamoxifen, a non-genomic ER ß agonist but selective antagonist of ER α, has been shown to have a beneficial effect on bone metabolism [11]. Tamoxifen may increase BMD in the lumbar spine and especially in post-menopausal women with breast cancer. However, little is known regarding its effect on fractures, and the results of previously published studies remain controversial in clinical practice. Cooke et al reported that current use of tamoxifen led to a significantly lower overall risk of osteoporotic fractures in a population-based case-control study [10]. However, a number of reports in the literature have reported no statistically significant bone protection at an age of 65 years and older [12, 13].

In comparison with previous population-based fracture risk studies, of which most have included post-menopausal woman, approximately 50 % of the cases were under 50 years of age in our study. Our results parallel another study in terms of the protective site of tamoxifen use. Tamoxifen has been shown to preserve BMD in the lumbar spine and femoral neck, and to protect against bone loss in the hip and spine but not the wrist in post-menopausal woman [14–17]. The preservation of BMD by tamoxifen in post-menopausal women is associated with a reduced risk of osteoporotic fractures.

Despite these findings, several studies have demonstrated that tamoxifen accelerates bone loss in pre-menopausal women. The results of the current study indicate that, after adjustment for potential confounding factors, patients receiving tamoxifen are at a lower risk of developing osteoporotic fractures overall, including spine and hip fractures, compared with those not receiving tamoxifen. The protective effect was also related to age, and especially those under 50 years of age. A lower risk of fractures was seen in both the older and younger patients. Another population-based study suggested that the protective effect of prior tamoxifen use was quickly lost after the treatment was changed to letrozole [10].

We did not compare the effect of other AIs with tamoxifen. Our results revealed that the protective effect was not related to the duration or dosage, so it may have been due to the steady usage of tamoxifen once a patient had been prescribed with this drug. There is a debate regarding the use of AIs and tamoxifen for the treatment of women with ER-positive breast cancer. The risk of relapse has been reported to persist after 5 years of treatment with adjuvant tamoxifen in post-menopausal women with hormone-sensitive early stage breast cancer [16]. Published cost-effectiveness analyses comparing AIs to tamoxifen have assumed an overall survival and indicated a higher risk of fractures with AIs, although this has not been shown in RCTs, leading to an overestimation of the cost-effectiveness of AIs [18]. If anastrozole use is associated with an increased risk of hip fractures, then the long-term benefits associated with this agent are reduced by approximately 25 % [19]. Less than half of hip fracture patients resume baseline levels of functioning, and nearly one in five requires long-term nursing home care after a hip fracture [20]. Concerns regarding the use of tamoxifen include menopausal symptoms, deep vein thrombosis, and endometrial cancer [21]. It has also been reported to increase the risk of abnormal endometrial thickness in post-menopausal women, although this has not been proven in pre-menopausal Asian women with breast cancer [22]. In addition, a recent study reported no increased risk of venous thromboembolism in Asian breast cancer patients receiving adjuvant tamoxifen treatment [23].

The strength of our study is the use of a population-based data set with the enrollment of a large number of patients. However, several limitations must be considered when interpreting these findings. The insurance claims files do not provide information on serum estrogen level, BMD, cancer stage or menopausal status. We were therefore unable to determine whether patients with advanced stages of the cancer or post-menopause were at a greater risk of fractures. Serum estrogen has been reported to be a risk factor for breast cancer, and a higher exposure to estrogen has been reported to preserve BMD and thereby decrease the risk of osteoporotic fractures [24–27]. A correlation between a higher BMD and a higher risk of breast cancer has been reported [28]. Therefore, the reduced risk of osteoporotic fractures in tamoxifen users may be due to prior exposure to endogenous estrogen [10].

Physicians need to weigh the benefits and risks of tamoxifen treatment in various clinical situations. In our opinion, the protective effect of tamoxifen is advised for younger patients, especially in Asian patients who have a higher risk of fractures at a younger age.
